# Predictive value of serum albumin-to-globulin ratio for incident chronic kidney disease: A 12-year community-based prospective study

**DOI:** 10.1371/journal.pone.0238421

**Published:** 2020-09-02

**Authors:** Jane Park, Hyung Jong Kim, Jinsu Kim, Yu Bum Choi, Yoon Soo Shin, Mi Jung Lee

**Affiliations:** 1 CHA University School of Medicine, CHA University, Seongnam, Korea; 2 Department of Internal Medicine, CHA Bundang Medical Center, CHA University, Seongnam, Korea; University of Liège, BELGIUM

## Abstract

**Background:**

Inflammation plays a pivotal role in the pathogenesis of chronic kidney disease (CKD). Significant association between serum albumin-to-globulin (AG) ratio and inflammation led us to investigate the prognostic value of serum AG ratio for incident CKD.

**Methods:**

The predictive value of serum AG ratio, white blood cell (WBC), and C-reactive protein (CRP) for CKD development was assessed in 8,057 non-CKD participants from a community-based, prospective cohort in Korea. Serum AG ratio was calculated by following equation: serum albumin (g/L)/[serum total protein (g/L)-serum albumin (g/L)]. Incident CKD was defined as estimated glomerular filtration rate <60 mL/min/1.73 m^2^ and/or proteinuria of more than 1+ on dipstick.

**Results:**

Median serum AG ratio was 1.38 (interquartile range, 1.28–1.52). During a mean follow-up duration of 9.1±3.7 years, 1,732 participants (21.5%) developed CKD. In a multivariable Cox analysis, a low serum AG ratio was significantly associated with an increased risk of incident CKD (Q1, serum AG ratio <1.26: hazard ratio [HR] = 1.651, 95% confidence interval [CI] = 1.406–1.938, Q5 as reference; per 0.2 decrease, HR = 1.170, 95% CI = 1.109–1.234). Serum AG ratio was the only indicator to improve the predictability of CKD development (net reclassification index = 0.158, P <0.001; integrated discrimination improvement = 0.005, P <0.001), compared with WBC or CRP.

**Conclusions:**

This study demonstrates that low serum AG ratio is an independent predictor for CKD development and exhibits a stronger predictive value than other inflammatory markers. These findings suggest that determining serum AG ratio may be more valuable for predicting adverse kidney outcomes in non-CKD populations.

## Introduction

The increasing incidence and prevalence of chronic kidney disease (CKD) had led to its global recognition as a preeminent public health problem [1]. Considering higher risk of cardiovascular complications and premature mortality caused by CKD, identifying the risk factors of CKD development would be useful for clinical practice. Apart from established traditional risk factors, chronic inflammation has been known to play a crucial role in the pathophysiology of CKD development [2–12]. To date, various inflammatory markers have been studied to determine their association to CKD development [4, 5, 7, 8, 10, 12]. Higher levels of inflammatory markers, including white blood cell (WBC), C-reactive protein (CRP), interleukin-6 (IL-6), and soluble tumor necrosis factor receptor 1 (sTNF-R1), are significantly associated with a greater risk of declining kidney function [4, 5, 7, 8, 10, 12].

Emerging evidence indicates that serum albumin-to-globulin ratio (AG ratio), calculated by dividing serum albumin by serum globulin (total protein-albumin), is an effective prognostic indicator in chronic diseases [13–19]. Although the mechanism by which serum AG ratio has a significant association with unfavorable clinical outcomes has yet to be fully clarified, chronic inflammation has been regarded as a convincing explanation [16, 19]. Low serum AG ratios have been observed in patients with rheumatoid arthritis and bronchiectasis, both of which are major chronic inflammatory diseases [16]. In a study of patients with systemic rheumatic diseases, serum AG ratio was correlated with CRP and erythrocyte sedimentation rate concentrations, suggesting the significant association of serum AG ratio with inflammation [20].

This significant association of serum AG ratio with chronic inflammation led us to hypothesize that serum AG ratio is a significant predictor of CKD development. However, to the best of our knowledge, the issue whether serum AG ratio associates with declining kidney function has not been explored before. Therefore, in the present study, we first investigated the prognostic value of serum AG ratio for CKD development using a large scale, community-based, prospective cohort in Korea. Furthermore, we compared the relative predictive value of serum AG ratio with other inflammatory markers to ascertain the clinical relevance of serum AG ratio in predicting kidney outcomes.

## Materials and methods

### Ethics statement

This study was conducted in accordance with the Declaration of Helsinki. The study protocol was approved by the ethics committee of the Korean Center for Disease Control and the institutional review board of CHA Bundang Medical Center (No. 2017-10-024). All participants provided written informed consent before enrollment.

### Study design and participants

This study analyzed participants enrolled in a community-based prospective cohort of the Korean Genome Epidemiology Study (KoGES), which comprises Korean general population aged 40–69 years who lived in the Ansung or Ansan communities from 2001 to 2002. Participants were followed up with every 2 years, and the latest (6^th^) follow-up was performed between 2013 and 2014. Detailed design and methods of the KoGES have been previously described [21]. Among the initially enrolled 10,300 participants, we excluded 1,973 participants and 8,057 non-CKD participants were finally analyzed (S1 Fig).

### Data collection and assessment of inflammatory markers

This study is supervised by the National Research Institute of Health, Korean Centers for Disease Control and Prevention. Data were retrieved from the electronic database with assistance from the Korean Centers for Disease Control. Demographics and medical history were collected by a trained research coordinator. Laboratory data were collected at baseline and every 2 years thereafter using a blood sample drawn after a 12-hr fast and the first voided urine. The protocols for sample collection, transportation, analysis, and storage have been established (www.cdc.go.kr). Collected blood and urine samples were refrigerated immediately and sent to the central laboratory of KoGES (Seoul Clinical Laboratories, Seoul, Korea) every day. All biochemical variables were measured at the central laboratory. Serum AG ratio was calculated using the following equation: serum AG ratio = Serum albumin (g/L)/[serum total protein (g/L)–serum albumin (g/L)]. WBC and CRP were selected as the two inflammatory markers to compare with serum AG ratio in this study. The Chronic Kidney Disease Epidemiology Collaboration equation was used to determine estimated glomerular filtration rate (eGFR) [22]. Urine dipstick test was performed using the first-voided urine, and proteinuria was categorized as absent, trace, 1+, 2+, or 3+.

### Follow-up and study outcome

Participants were followed up with every 2 years by site visits. Among the 8,057 study participants, 4,145 (51.4%) completed all baseline and six follow-up visit. The study outcome was the development of CKD, defined by an eGFR of <60mL/min/1.73 m^2^ and/or proteinuria of more than 1+ on dipstick test, whichever occurred first. Patients who were lost to follow up were censored at the date of the last examination (444 at the 1st, 464 at the 2nd, 301 at the 3rd, 364 at the 4th, and 576 at the 5th follow-up visit).

### Statistical analysis

Statistical analysis was performed using R (R Foundation for Statistical Computing, Vienna, Austria; www.r-project.org). Continuous variables were expressed as the means ± standard deviation (SD) or the medians (interquartile range [IQR]), and categorical variables were expressed as raw numbers (percentages). Participants were categorized into quintiles by the following serum AG ratio values: Q1, <1.26; Q2, 1.26 to <1.34; Q3, 1.34 to <1.42; Q4, 1.42 to <1.55; and Q5, ≥1.55. Baseline characteristics were compared among the five groups using ANOVA with Bonferroni *post hoc* test, or the Kruskal-Wallis test for continuous variables, and the chi-square test for categorical variables. The association of serum AG ratio with other inflammatory markers was evaluated using Spearman’s rank test. Furthermore, binary logistic regression analyses were performed to explore significant variables associated with low serum AG ratio (Q1 and Q2, AG ratio <1.34). To identify the independent prognostic value of serum AG ratio for CKD development, multivariable Cox models were constructed including significant variables from the univariate analyses. In addition, the prognostic value of serum AG ratio was evaluated in a fractional polynomial model. Sensitivity analyses were performed for other renal outcomes, including development of proteinuria, eGFR of <60 ml/min/1.73 m^2^, eGFR decline of >50% from baseline, and rapid decline of kidney function (eGFR decline >3 ml/min/1.73 m^2^ per year). Subgroup analyses according to age (40 to 49 years or 50 to 69 years), sex, smoking status (smokers or never smokers), and eGFR categories (≥90 ml/min/1.73 m^2^ or 60 to 89 ml/min/1.73 m^2^) were also performed. Moreover, to mitigate confounding effect of nutritional status, muscle mass, acute infection, or chronic inflammatory diseases subgroup analyses excluding underweight participants (body mass index [BMI] <18.5 kg/m^2,^ 144 [1.8%] participants), participants who their CRP concentrations were above 95 percentile (CRP >0.66 mg/L, 403 [5.0%] participants), or participants with eGFR of <70 ml/min/1.73 m^2^ (487 [6.0%] participants) were also performed. To compare the relative predictive value of serum AG ratio, WBC, and CRP for CKD development, we assessed the additional effect of each inflammatory indicator on the base model: model 1, base model + serum AG ratio; model 2, base model + WBC; and model 3, base model + CRP. The area under the receiver operating curve (AUC), *c* Statistics, the net reclassification index (NRI) and the integrated discrimination improvement (IDI) were calculated to ascertain which inflammatory indicators improved discriminatory ability when added to the base model. A P value less than 0.05 was considered statistically significant.

## Results

### Baseline characteristics according to the serum AG ratio categories

Baseline characteristics according to serum AG ratio categories are shown in Table 1. Of the 8,057 participants included in the study, 3,872 (48.1%) were men. The mean age of study participants was 51.7±8.7 years. For all participants, the median of AG ratio was 1.38 (IQR, 1.28–1.52), and the median AG ratio for each quintile, Q1-Q5 were 1.19, 1.30, 1.38, 1.48, and 1.66, respectively. Age, BMI, CRP, and eGFR significantly decreased as the AG ratio increased. By contrast, hemoglobin, cholesterol, and glucose concentrations significantly increased as the AG ratio increased. Participants with a higher AG ratio were more likely to be men or living in a high-income household.

**Table 1:** Baseline demographics and clinical characteristics of participants according to the serum AG ratio categories.

	All	Quintile 1	Quintile 2	Quintile 3	Quintile 4	Quintile 5	
		(<1.26)	(1.26 to <1.34)	(1.34 to <1.42)	(1.42 to <1.55)	(≥1.55)	
	(*n* = 8,057)	(*n* = 1,643)	(*n* = 1,643)	(*n* = 1,483)	(*n* = 1,636)	(*n* = 1,652)	P
Serum AG ratio	1.38 (1.28–1.52)	1.19 (1.14–1.22)	1.30 (1.28–1.32)	1.38 (1.37–1.41)	1.48 (1.45–1.52)	1.66 (1.59–1.76)	<0.001
Age, years	51.7 ± 8.7	53.0 ± 8.8	52.0 ± 8.7	51.8 ± 8.7	51.4 ± 8.6	50.2 ± 8.5	<0.001
Men, *n* (%)	3,872 (48.1%)	512 (31.2%)	640 (39.0%)	666 (44.9%)	922 (56.4%)	1,132 (68.5%)	<0.001
Education, *n* (%)							<0.001
≤6th grade	2,537 (31.5%)	659 (40.1%)	583 (35.5%)	451 (30.4%)	455 (27.8%)	389 (23.5%)	
7th-12th grade	4,408 (54.7%)	822 (50.0%)	890 (54.2%)	859 (57.9%)	913 (55.8%)	924 (55.9%)	
>12th grade	1,112 (13.8%)	162 (9.9%)	170 (10.3%)	173 (11.7%)	268 (16.4%)	339 (20.5%)	
Income, *n* (%)							<0.001
<$1,000/m	2,707 (33.6%)	678 (41.3%)	603 (36.7%)	521 (35.1%)	483 (29.5%)	422 (25.5%)	
$1,000 to $2,000/m	2,398 (29.8%)	471 (28.7%)	507 (30.9%)	449 (30.3%)	506 (30.9%)	465 (28.1%)	
>$2,000/m	2,952 (36.6%)	494 (30.1%)	533 (32.4%)	513 (34.6%)	647 (39.5%)	765 (46.3%)	
Smokers, *n* (%)	3,288 (40.9%)	399 (24.2%)	525 (32.0%)	564 (38.0%)	790 (48.3%)	1,010 (61.1%)	<0.001
Regular exercises, *n* (%)	2,299 (28.5%)	445 (27.1%)	446 (27.1%)	420 (28.3%)	464 (28.4%)	524 (31.7%)	0.02
Comorbid disease, *n* (%)							
Diabetes mellitus	335 (4.2%)	80 (4.9%)	58 (3.5%)	43 (2.9%)	74 (4.5%)	80 (4.8%)	0.02
Hypertension	1,159 (14.4%)	275 (16.7%)	245 (14.9%)	208 (14.0%)	210 (12.8%)	221 (13.4%)	0.01
CAD	59 (0.7%)	13 (0.8%)	13 (0.8%)	10 (0.7%)	8 (0.5%)	15 (0.9%)	0.69
PAD	23 (0.3%)	4 (0.2%)	5 (0.3%)	5 (0.3%)	5 (0.3%)	4 (0.2%)	0.9
CVA	79 (1.0%)	12 (0.7%)	11 (0.7%)	12 (0.8%)	20 (1.2%)	24 (1.5%)	0.09
CHF	16 (0.2%)	4 (0.2%)	4 (0.2%)	5 (0.3%)	3 (0.2%)	0 (0%)	0.28
^a^CVD	225 (2.8%)	52 (3.2%)	40 (2.4%)	37 (2.5%)	41 (2.5%)	55 (3.3%)	0.36
Liver disease	359 (4.5%)	96 (5.8%)	69 (4.2%)	60 (4.0%)	68 (4.2%)	66 (4.0%)	0.05
BMI, kg/m^2^	24.6 ± 3.1	25.0 ± 3.3	24.8 ± 3.2	24.5 ± 3.2	24.4 ± 3.0	24.2 ± 3.0	<0.001
SBP, mmHg	120.9 ± 18.1	122.4 ± 18.6	121.9 ± 18.7	120.8 ± 19.1	119.9 ± 17.3	119.4 ± 16.7	<0.001
DBP, mmHg	80.1 ± 11.4	80.2 ± 11.4	80.1 ± 11.5	79.9 ± 11.8	79.7 ± 11.2	80.6 ± 11.1	0.21
Hemoglobin, g/dL	13.6 ± 1.6	13.2 ± 1.6	13.4 ± 1.5	13.5 ± 1.6	13.8 ± 1.5	14.1 ± 1.5	<0.001
WBC, x10^3^/uL	6.5 ± 1.8	6.7 ± 1.9	6.5 ± 1.8	6.4 ± 1.7	6.5 ± 1.8	6.5 ± 1.7	0.001
BUN, mg/dL	14.2 ± 3.5	13.9 ± 3.5	14.1 ± 3.5	14.1± 3.5	14.5 ± 3.6	14.6 ± 3.5	<0.001
Creatinine, mg/dL	0.8 ± 0.2	0.7 ± 0.1	0.8 ± 0.1	0.8 ± 0.2	0.9 ± 0.2	0.9 ± 0.2	<0.001
Total Protein, g/dL	7.3 ± 0.4	7.5 ± 0.4	7.3 ± 0.4	7.2 ± 0.4	7.2 ± 0.5	7.3 ± 0.5	<0.001
Albumin, g/dL	4.3 ± 0.3	4.1 ± 0.2	4.1 ± 0.2	4.2 ± 0.2	4.3 ± 0.3	4.6 ± 0.3	<0.001
Glucose, mg/dL	87.0 ± 20.5	85.7 ± 22.2	85.8 ± 19.5	85.3 ± 18.2	86.8 ± 18.4	91.1 ± 22.9	<0.001
Total cholesterol, mg/dL	191.2 ± 34.7	185.7 ± 34.7	187.9 ± 33.0	188.5 ± 33.2	194.0 ± 35.3	199.6 ± 35.4	<0.001
CRP, mg/L	0.14 (0.06–0.24)	0.17 (0.08–0.30)	0.15 (0.08–0.25)	0.13 (0.06–0.22)	0.13 (0.06–0.23)	0.11 (0.04–0.20)	<0.001
eGFR, mL/min/1.73 m^2^	93.0 ± 13.1	94.5 ± 12.1	94.6 ± 12.1	94.4 ± 12.6	91.8 ± 13.5	89.7 ± 14.1	<0.001

Data are expressed as the mean ± standard deviation, median (interquartile range), or number of patients (percent).

^a^CVD: A composite of CAD, PAD, CVA, and CHF.

*Abbreviations*: AG ratio, albumin-to-globulin ratio; BMI, body mass index; CAD, coronary artery disease; CHF, congestive heart failure; CRP, C-reactive protein; CVA, cerebrovascular accident; CVD, cardiovascular disease; DBP, diastolic blood pressure; eGFR, estimated glomerular filtration rate; PAD, peripheral artery disease; SBP, systolic blood pressure; WBC, white blood cell.

### Significant variables associated with low serum AG ratio

Serum AG ratio significantly associated with WBC counts (*r* = –0.04; P = 0.002) and CRP concentrations (*r* = –0.15; P <0.001). Furthermore, binary logistic regression analysis for low serum AG ratio (Q1 and Q2, AG ratio <1.34) showed that higher WBC counts (per 1.8x10^3^/uL increase; odds ratio, 1.166; 95% confidence interval [CI], 1.110–1.225) and higher CRP concentrations (per 0.46 mg/L increase; odds ratio, 1.171; 95% CI, 1.097–1.250) were independently associated with low AG ratio (S1 Table). Moreover, old age, higher levels of systolic blood pressure and baseline eGFR, lower concentrations of hemoglobin and total cholesterol, women, non-smokers, and obesity were also significantly associated with low AG ratio.

### Serum AG ratio as an independent predictor of incident CKD

**Fig 1:**
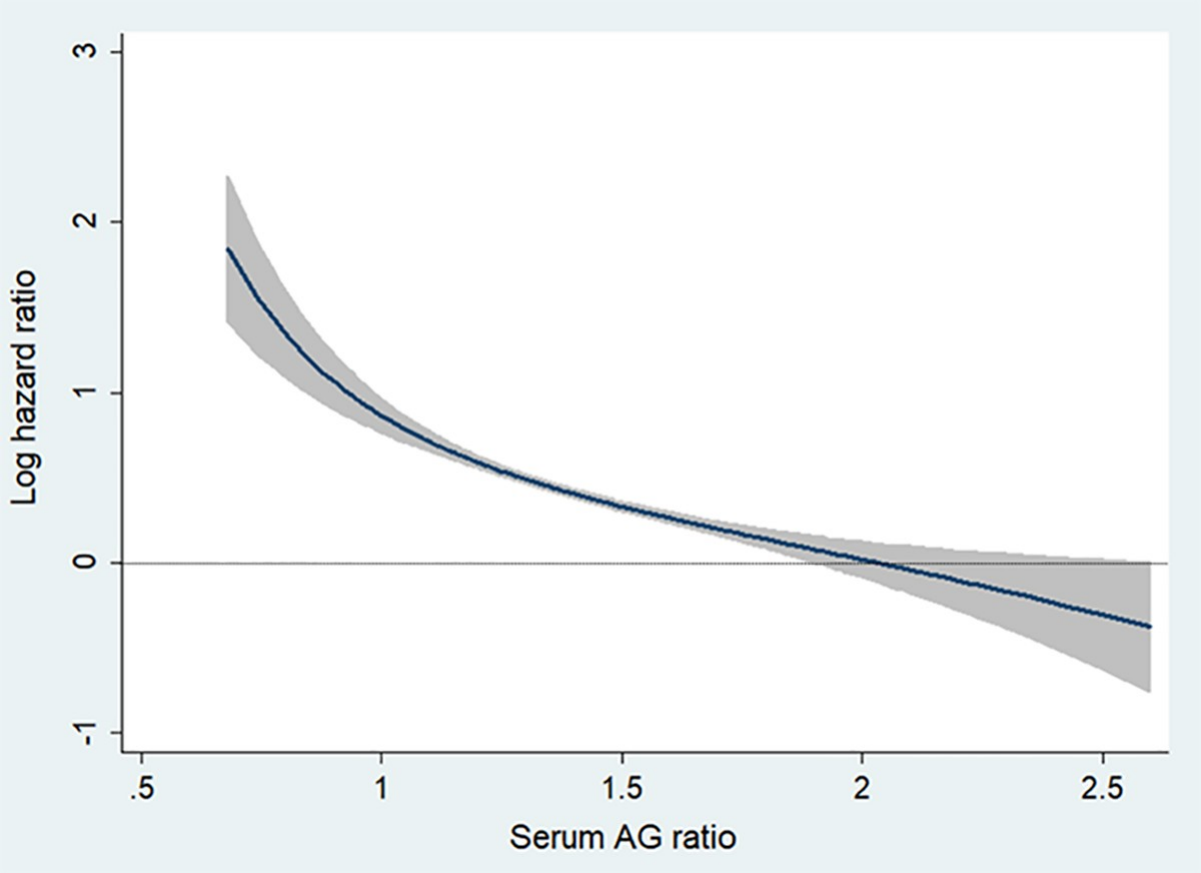
Multivariate fractional polynomial graphs for association between serum AG ratio and incident CKD. Log hazard ratios were calculated after adjustment for age, sex, education and income levels, smoking status, diabetes, hypertension, cardiovascular disease, body mass index, mean arterial pressure, hemoglobin, serum glucose, total cholesterol, and baseline eGFR. Shaded areas indicate 95% confidence limits. *Abbreviations*: AG ratio, albumin-to-globulin ratio; CKD, chronic kidney disease; eGFR, estimate glomerular filtration rate.

During a mean follow-up duration of 9.1±3.7 years, CKD developed in 1,732 participants (21.5%) (Table 2). The incidence rate of CKD was the lowest in Q5 (16.8%) and the highest in Q1 (26.5%) of serum AG ratio. To determine the independent association of serum AG ratio and incident CKD, multivariable Cox regression analysis was performed (Table 2, S2 and S3 Tables). Compared to those in the reference group (Q5), participants in serum AG ratio groups Q1-Q4 had the following hazard ratios (HRs) for CKD development: Q1, 1.651 (95% CI, 1.406–1.938; P<0.001); Q2, 1.453 (95% CI, 1.235–1.708; P<0.001); Q3, 1.419 (95% CI, 1.203–1.675; P<0.001); Q4, 1.174 (95% CI, 0.998–1.381; P = 0.05). When serum AG ratio was analyzed as a continuous variable, low serum AG ratio was significantly associated with risk of incident CKD (per 0.2 decrease: HR, 1.170; 95% CI, 1.109–1.234; P <0.001). Moreover, fractional polynomial analysis also showed that the risk of incident CKD decreased steadily with higher serum AG ratio values (Fig 1). In contrast, WBC and CRP showed conflicting results depending on whether they were analyzed as categorical or continuous variables (Table 2). Higher WBC was only significantly associated with CKD development when analyzed as a continuous variable (per 1.8x10^3^/uL increase: HR, 1.051; 95% CI, 1.002–1.102; P = 0.04). However, CRP was only significant associated with CKD development when analyzed as categorical variable (Q1 as reference; Q5, HR, 1.051; 95% CI, 1.002–1.102; P = 0.04).

**Table 2:** Hazard ratios of serum AG ratio, WBC, and CRP categories for CKD development.

		Crude		^a^Fully adjusted	
	*n* (%)	HR (95% CI)	P	HR (95% CI)	P
Serum AG ratio quintiles					
Q1 (<1.26)	436 (26.5%)	1.608 (1.384–1.870)	<0.001	1.651 (1.406–1.938)	<0.001
Q2 (1.26 to <1.34)	379 (23.1%)	1.328 (1.138–1.551)	<0.001	1.453 (1.235–1.708)	<0.001
Q3 (1.34 to <1.42)	317 (21.4%)	1.253 (1.066–1.472)	0.006	1.419 (1.203–1.675)	<0.001
Q4 (1.42 to <1.55)	323 (19.7%)	1.141 (0.972–1.340)	0.11	1.174 (0.998–1.381)	0.05
Q5 (≥1.55)	277 (16.8%)	1 (reference)		1 (reference)	
Serum AG ratio (per 0.2 decrease)		1.166 (1.108–1.226)	<0.001	1.170 (1.109–1.234)	<0.001
WBC quintiles, x10^3^/uL					
Q1 (<5.1)	349 (19.7%)	1 (reference)		1 (reference)	
Q2 (5.1 to <5.9)	330 (20.9%)	1.082 (0.930–1.257)	0.31	1.017 (0.874–1.184)	0.83
Q3 (5.9 to <6.7)	366 (22.9%)	1.210 (1.045–1.401)	0.01	1.156 (0.996–1.342)	0.06
Q4 (6.7 to <7.8)	325 (21.0%)	1.104 (0.949–1.284	0.20	1.032 (0.884–1.205)	0.69
Q5 (≥7.8)	362 (23.2%)	1.251 (1080–1.449)	0.003	1.160 (0.996–1.350)	0.06
WBC (per 1.8x10^3^/uL increase)		1.076 (1.028–1.126)	0.002	1.051 (1.002–1.102)	0.04
CRP quintiles, mg/L					
Q1 (<0.05)	321 (17.9%)	1 (reference)		1 (reference)	
Q2 (0.05 to <0.110)	277 (21.3%)	1.005 (0.856–1.181)	0.9	0.941 (0.800–1.105)	0.46
Q3 (0.110 to <0.170)	334 (21.3%)	1.248 (1.070–1.454)	0.01	1.147 (0.983–1.338)	0.08
Q4 (0.170 to <0.280)	383 (24.1%)	1.394 (1.202–1.617)	<0.001	1.162 (1.000–1.352)	0.51
Q5 (≥0.280)	417 (27.2%)	1.689 (1.460–1.953)	<0.001	1.243 (1.171–1.443)	0.004
CRP (per 0.46 mg/L increase)		1.037 (1.011–1.063)	0.01	1.001 (0.960–1.043)	0.9

^a^Fully adjusted: adjusted for age, sex, education and income levels, smoking status, diabetes, hypertension, cardiovascular disease, body mass index, mean arterial pressure, hemoglobin, serum glucose, total cholesterol, and baseline eGFR.

*Abbreviations*: AG ratio, albumin-to-globulin ratio; CI, confidence interval; CKD, chronic kidney disease; CRP, C-reactive protein; eGFR, estimated glomerular filtration rate; HR, hazard ratio; WBC, white blood cell.

### Sensitivity analysis

**Fig 2:**
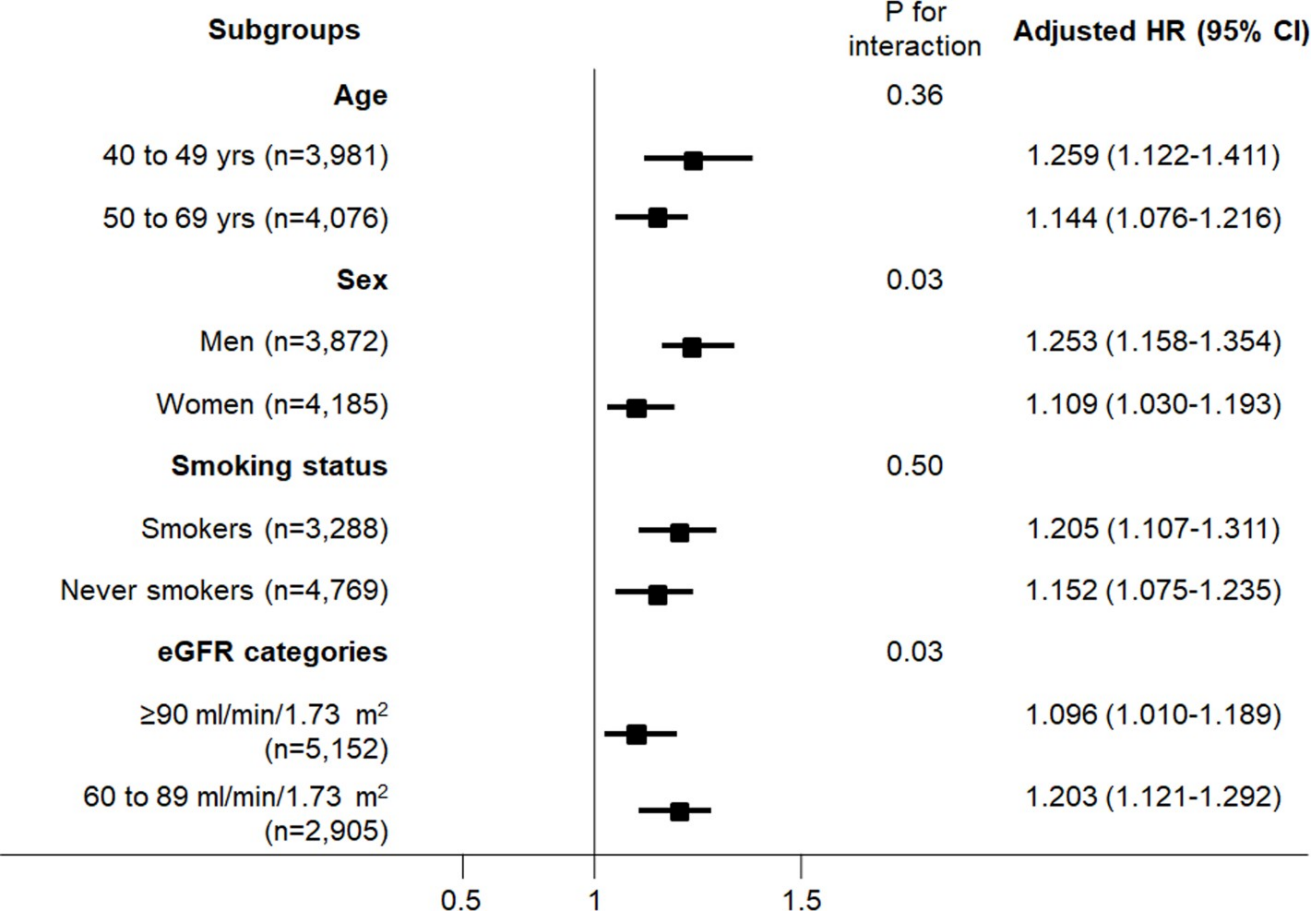
Fully adjusted HRs of low serum AG ratio (per 0.2 decrements) for incident CKD according to age, sex, smoking status, and eGFR subgroups. Fully adjusted HRs were calculated after adjustment for age, sex, education and income levels, smoking status, diabetes, hypertension, cardiovascular disease, body mass index, mean arterial pressure, hemoglobin, serum glucose, total cholesterol, and baseline eGFR. *Abbreviations*: AG ratio, albumin-to-globulin ratio; CI, confidence interval; CKD, chronic kidney disease; eGFR, estimated glomerular filtration rate; HR, hazard ratio.

Sensitivity analyses were performed for other renal outcomes (S4 Table and Table 3). Low serum AG ratio (Q1; AG ratio <1.26) was consistently associated with other adverse renal outcomes (proteinuria; HR,1.922; 95% CI,1.285–2.875; eGFR of <60 ml/min/1.73 m^2^; HR, 1.648; 95% CI, 1.391–1.953; eGFR decline of >50% from baseline; HR, 2.064; 95% CI, 1.073–3.971; rapid decline of kidney function; odds ratio, 1.375; 95% CI, 1.199–1.578). Subgroup analyses according to age, sex, smoking status, and eGFR categories also showed the significant association of low serum AG ratio and incident CKD (Fig 2). Moreover, the association between low serum AG ratio and CKD development remained significant in subgroup analyses excluding participants with underweight (S5 Table), high CRP concentrations (S6 Table), or eGFR of <70 ml/min/1.73 m^2^ (S7 Table).

**Table 3:** Fully adjusted risks of serum AG ratio for various kidney outcomes.

	^a^Proteinuria		eGFR of <60 ml/min/1.73 m^2^		eGFR decline of >50% from baseline		^b^Rapid decline of kidney function	
	HR (95% CI)	P	HR (95% CI)	P	HR (95% CI)	P	OR (95% CI)	P
Serum AG ratio quintiles								
Q1 (<1.26)	1.922 (1.285–2.875)	0.001	1.648 (1.391–1.953)	<0.001	2.064 (1.073–3.971)	0.03	1.375 (1.199–1.578)	<0.001
Q2 (1.26 to <1.34)	1.367 (0.901–2.075)	0.14	1.489 (1.254–1.767)	<0.001	1.493 (0.748–2.981)	0.26	1.177 (1.023–1.354)	0.02
Q3 (1.34 to <1.42)	1.337 (0.874–2.045)	0.18	1.440 (1.208–1.717)	<0.001	2.458 (1.255–4.817)	0.01	1.201 (1.041–1.386)	0.01
Q4 (1.42 to <1.55)	1.239 (0.829–1.853)	0.30	1.136 (0.954–1.351)	0.15	1.639 (0.815–3.294)	0.17	1.086 (0.940–1.255)	0.26
Q5 (≥1.55)	1 (reference)		1 (reference)		1 (reference)		1 (reference)	
Serum AG ratio (per 0.2 decrease)	1.229 (1.071–1.410)	0.003	1.178 (1.113–1.247)	<0.001	1.186 (0.983–1.431)	0.07	1.105 (1.056–1.156)	<0.001

Fully adjusted hazard ratios were calculated after adjustment for age, sex, education and income levels, smoking status, diabetes, hypertension, cardiovascular disease, body mass index, mean arterial pressure, hemoglobin, serum glucose, total cholesterol, and baseline eGFR.

^a^Proteinuria was defined as urinary protein of more than 1+ on dipstick tes

^b^Rapid decline of kidney function was defined as eGFR decline >3 ml/min/1.73 m^2^ per year.

*Abbreviations*: AG ratio, albumin-to-globulin ratio; CI, confidence interval; eGFR, estimated glomerular filtration rate; HR, hazard ratio; OR, odds ratio.

### Comparison of serum AG ratio, WBC, and CRP for predicting incident CKD

To compare the additive predictive value of serum AG ratio and other inflammatory markers to the base model for incident CKD, AUC, *c* Statistics, NRI and IDI were calculated (Table 4 and S8 Table). Only serum AG ratio significantly improved the predictive value for incident CKD compared with the base model (AUC = 0.7810, P = 0.01 compared to base model; *c* Statistics = 0.776, P = 0.004 compared to base model; continuous NRI = 0.158, P <0.001; categorical NRI (<10%, 10–50%, >50%) = 0.027, P<0.001 IDI = 0.005, P <0.001). When WBC and CRP were added to the base model, no significant improvements in predicting CKD development were observed.

**Table 4:** Predictability of serum AG ratio, WBC, and CRP for CKD development using *c* statistics, AUC, NRI, and IDI.

Models	AUC	^a^P	*c* Statistics	^a^P	NRI (SEM; 95% CI)	P	IDI (SEM; 95% CI)	P
^b^Base model	0.7782		0.774		Reference		Reference	
Base model + AG ratio	0.7810	0.01	0.776	0.004	0.158 (0.105–0.210)	<0.001	0.005 (0.003–0.007)	<0.001
Base model + WBC	0.7784	0.67	0.775	0.35	0.041 (-0.011, 0.094)	0.13	0.001 (0, 0.001)	0.07
Base model + CRP	0.7781	0.61	0.774	0.73	-0.002 (-0.050, 0.047)	0.9	0.001 (-0.001, 0.001)	0.75

^a^Compared to base model

^b^Base model included age, sex, education and income levels, smoking status, diabetes, hypertension, cardiovascular disease, body mass index, mean arterial pressure, hemoglobin, serum glucose, total cholesterol, and baseline eGFR.

*Abbreviations*: AG ratio, albumin-to-globulin ratio; AUC, area under the receiver operating curve; CI, confidence interval; CKD, chronic kidney disease; CRP, C-reactive protein; eGFR, estimated glomerular filtration rate; IDI, integrated discrimination improvement; NRI, net reclassification index; SEM, standard error of the means; WBC, white blood cell.

## Discussion

This study demonstrates that low serum AG ratio is a significant predictor of CKD development in the general Korean population. Furthermore, serum AG ratio was the only indicator to improve the discriminative power of the base model for predicting CKD development, compared to well-known inflammatory markers, WBC and CRP.

Because medical and socioeconomic burdens for patients with CKD have increased over time, a method for identifying people who are at high risk of CKD development would be useful for research and clinical practice. Besides well-known traditional risk factors such as diabetes and hypertension, inflammation has been regarded as one of the key underlying factors in the development of CKD [2–12]. Moreover, significant associations between traditional risk factors and CKD are implicated in the inflammatory process [11]. Several previous studies have demonstrated that chronic inflammation is prevalent in CKD patients, and this prevalence increases as kidney function decreases [11]. So far, various inflammatory markers have been evaluated by stratifying their association with CKD development in non-CKD populations [4, 5, 7, 8, 10, 12]. Elevated WBC was significantly associated with incident CKD, defined as treatment for kidney failure or death related to kidney disease [4, 5]. The Multi-Ethnic Study of Atherosclerosis revealed that higher IL-6 and CRP concentrations indicate a faster declining rate of kidney function [8]. In a cross-sectional and observational analysis of a population-based cohort, various inflammatory markers such as WBC, high-sensitivity CRP, IL-6, and sTNF-R1 were positively associated with CKD prevalence [7]. In the present study, higher WBC and the highest quintile of CRP indicated an increased risk of CKD development, suggesting that inflammation significantly affects kidney injury in accordance with previous studies [4, 5, 8]. Based on these findings, we surmised that although various inflammatory markers can be used for predicting incident CKD risk, each marker should be independently verified.

Low AG ratio was initially studied as a clinical indicator for multiple myeloma and other immunoproliferative diseases [23–25]. Theoretically, low AG ratio can be derived from high globulin, low albumin, or both. High globulin is found in cancer, rheumatoid diseases, and chronic liver disease, and low albumin is associated with chronic infections, malnutrition, chronic liver disease, and nephrotic syndrome [16, 23–31]. Emerging evidence has demonstrated that low AG ratio is implicated in adverse clinical outcomes, in association with chronic inflammation [13–19]. Low AG ratio is a significant predictor of mortality in breast, lung, colorectal and kidney cancer [13–15, 18]. He et al. [19] indicated that higher AG ratio (cut-off, 1.15–1.75) is correlated with better overall survival, cancer-specific survival, disease-free survival, and disease-metastasis-free survival in a recent meta-analysis of 13,890 solid cancer patients from 24 studies. They suggested that chronic inflammation, which is involved in all kinds of serum globulin, contributes to tumor proliferation, immune evasion, and metastasis [19]. Low serum AG ratios are frequently observed in patients with rheumatoid arthritis and bronchiectasis, both of which are major chronic inflammatory diseases [16]. In patients with systemic rheumatic diseases, serum AG ratio was correlated with CRP and erythrocyte sedimentation rate concentrations, supporting the significant association of serum AG ratio with inflammation [20]. Given that metabolic alteration caused by chronic diseases and chronic inflammation are implicated in CKD development, we hypothesized that serum AG ratio may be useful for predicting the risk of declining kidney function. In the present study, higher WBC counts and CRP concentrations were independently associated with low serum AG ratio. Furthermore, low serum AG ratio was an independent indicator for predicting an increased risk of incident CKD. Although the underlying mechanism of the significant association between low serum AG ratio and declining kidney function has not been investigated, we surmised that inflammation can be one of the possible explanations. Inflammatory disease and other illnesses reduced serum albumin concentrations via the down-regulation of albumin mRNA by the liver, increased albumin catabolism, and vascular permeability [32–34], leading to low albumin concentrations, one component of a low AG ratio. Moreover, chronic inflammation increased serum combined immunoglobulin free light chain excessively, which leads to high globulin concentrations, the second component of a low AG ratio [26, 29–31]. Evidence from several previous in vitro and in vivo studies support our speculation that inflammatory processes may play a pivotal role in the development of CKD [2, 3, 6, 7, 9]. However, albumin concentrations can be affected by nutritional status or muscle mass. In the current study, old age, women, higher baseline eGFR, and lower concentrations of hemoglobin and total cholesterol were significantly associated with low AG ratio, suggesting the effect of under-nutrition or low muscle mass. Although subgroup analyses excluding participants with underweight or high CRP concentration consistently showed a significant association of low AG ratio and incident CKD, we cannot totally mitigate confounding effects. Therefore, our findings should be interpreted with caution and further studies are needed to elucidate the underlying mechanism for the association between low AG ratio and adverse renal outcomes.

In this study, serum AG ratio had a greater predictive value for incident CKD than WBC and CRP. Of note, serum AG ratio was the only indicator to improve the discriminative ability of the base model for predicting incident CKD. In a retrospective analysis of 592 patients with renal cell carcinoma, the prognostic value of serum AG ratio was higher than those of other established inflammation-based prognostic scores, including neutrophil to lymphocyte ratio, monocyte to lymphocyte ratio, and Glasgow prognostic score, in accordance with our finding [18]. However, we did not assess other inflammatory markers such as IL-6 and sTNF-R1 and cannot compare the relative predictive value of these markers. Although the prognostic value of IL-6 and sTNF-R1 as inflammatory markers is well-known [7, 8, 10], these markers are relatively expensive and require additional sampling, both of which limit their use in routine clinical practice. In contrast, the method of measuring serum total protein and albumin is a simple and cost-effective strategy for assessing and improving public health. Based on these findings, we suggest that this simple method of measuring serum AG ratio has a greater clinical advantage for predicting adverse kidney outcomes than measuring other inflammatory markers in non-CKD population. Furthermore, serum AG ratio may be more valuable than other markers for translating study results to real world clinical practice, especially in large-scale cohort studies.

This study has several limitations. First, because all blood and urine samples were analyzed at the central laboratory for pre-specified biochemical variables, direct measurement of globulin was not available. In this study, both low serum albumin and total protein were significantly associated with risk of incident CKD (data not shown). Therefore, we cannot clarify the issue whether albumin or globulin is a main contributor of AG ratio without measuring globulin. Second, because this study was an observational cohort study, we cannot evaluate the causal relationship of serum AG ratio with CKD development. Furthermore, our study does not include any information regarding intervention of serum AG ratio to prevent CKD development. A community based cohort study including general population was not suitable to address therapeutic potential of serum AG ratio for adverse kidney outcomes. Third, although we surmised that inflammation may contribute to the significant association between serum AG ratio and adverse kidney outcome, this study could not clarify the underlying mechanism of this association. Therefore, further studies should be performed to better elucidate this mechanism. Fourth, although our study participants were generally healthy adults from a community-based cohort, we cannot guarantee no confounding effect due to acute infection or malnutrition influenced serum albumin and globulin concentrations. In this regard, determining kidney function using creatinine based eGFR may have a bias due to the effect of muscle mass. Fifth, we did not confirm the development of CKD using a second test of eGFR or urinalysis. Because data for death, initiating dialysis or performing kidney transplantation were not available, we cannot evaluate competing risk or end-stage renal disease risk. To alleviate this limitation, additional renal outcomes, including eGFR decline of >50% from baseline and rapid decline of kidney function, were tested, the significant association of serum AG ratio with these outcomes was consistent. Sixth, serum AG ratio was determined once at baseline and was not measured serially during follow-up. Thus, we cannot examine whether changes in serum AG ratio during the follow-up period exert any influence on kidney function. Lastly, because study participants were all Korean non-CKD adults, the predictive value of serum AG ratio may not be generalized to other populations. Large prospective studies in other populations should be conducted to verify our findings. Notwithstanding these limitations, this study also has specific clinical strengths. This study for the first time revealed the prognostic value of serum AG ratio for incident CKD and ascertained the advantage of serum AG ratio in predicting CKD over other inflammatory markers. This study’s large sample size of non-CKD participants from a well-examined community-based cohort provided high-quality data, and the long follow-up of 12 years was sufficient to ascertain adverse kidney outcomes in a non-CKD population.

In conclusion, low serum AG ratio is a significant predictor of CKD development and the only indicator to improve the predictive ability for incident CKD compared with other inflammatory markers in a non-CKD population. These findings suggest that serum AG ratio may be more valuable by stratifying the risk of adverse kidney outcomes in this population.

## Supporting information

S1 Table Binary logistic regression analysis of variables for low serum AG ratio. (PDF)

S2 Table Crude and fully adjusted hazard ratios of serum AG ratio quintiles and other variables for CKD development. (PDF)

S3 Table Crude and fully adjusted hazard ratios of serum AG ratio (per 0.2 decrement) and other variables for CKD development. (PDF)

S4 Table The number of participants with various kidney outcomes according to serum AG ratio quintiles. (PDF)

S5 Table Fully adjusted hazard ratios of serum AG ratio for CKD development after excluding underweight participants. (PDF)

S6 Table Fully adjusted hazard ratios of serum AG ratio for CKD development after excluding participants with high CRP concentrations. (PDF)

S7 Table Fully adjusted hazard ratios of serum AG ratio for CKD development after excluding participants with eGFR of <70 ml/min/1.73 m^2^. (PDF)

S8 Table Reclassification table and categorical NRI of base model, serum AG ratio, WBC, and CRP for CKD development. (PDF)

S1 Fig Flow diagram of participants. (TIF)
