# Supplementary material for: Predictive value of serum albumin-to-globulin ratio for incident chronic kidney disease: A 12-year community-based prospective study
Source: PLoS One. 2020 Sep 2;15(9):e0238421. doi: 10.1371/journal.pone.0238421 (PMC7467286; doi:10.1371/journal.pone.0238421)
Supplement: S1 Table — (PDF) [file pone.0238421.s001.pdf]

S1 Table. Binary logistic regression analysis of variables for low serum AG ratio

	Crude		Adjusted	
	OR (95% CI)	P	OR (95% CI)	P
Age (per year)	1.018 (1.012-1.023)	<0.001	1.021 (1.013-1.028)	<0.001
Women (vs. men)	2.457 (2.241-2.693)	<0.001	1.377 (1.155-1.641)	<0.001
Education ≥7th grade (vs. <7th grade)	0.613 (0.558-0.674)	<0.001	0.946 (0.837-1.070)	0.38
Income ≥$1,000/m (vs. <$1,000/m)	0.667 (0.608-0.733)	<0.001	0.898 (0.799-1.010)	0.07
Smoker (vs. never smoker)	0.398 (0.362-0.438)	<0.001	0.563 (0.485-0.653)	<0.001
Diabetes Mellitus (vs. no)	1.018 (0.816-1.271)	0.88	1.276 (0.982-1.657)	0.07
Hypertension (vs. no)	1.216 (1.072-1.378)	0.002	0.942 (0.813-1.092)	0.43
BMI groups (kg/m^2)				
Underweight (<18.5)	1 (reference)		1 (reference)	
Normal (18.5 to 22.9)	0.947 (0.670-1.339)	0.76	1.012 (0.706-1.449)	0.9
Overweight (23 to 24.9)	1.048 (0.741-1.484)	0.79	1.174 (0.818-1.687)	0.39
Obese (≥25)	1.294 (0.919-1.823)	0.14	1.461 (1.019-2.095)	0.04
SBP (per mmHg)	1.006 (1.004-1.009)	<0.001	1.004 (1.001-1.007)	0.01
Hemoglobin (per mg/dL)	0.790 (0.766-0.813)	<0.001	0.942 (0.901-0.984)	0.01
Glucose (per mg/dL)	0.995 (0.992-0.997)	<0.001	0.997 (0.995-1.000)	0.07
Total cholesterol (per mg/dL)	0.994 (0.992-0.995)	<0.001	0.994 (0.992-0.995)	<0.001
eGFR (per $mL/min/1.73m^2$)	1.016 (1.013-1.020)	<0.001	1.018 (1.014-1.022)	<0.001
WBC (per $1.8x10^3/uL$)	1.066 (1.020-1.114)	0.01	1.166 (1.110-1.225)	<0.001
CRP (per 0.46 mg/L)	1.186 (1.115-1.262)	<0.001	1.171 (1.097-1.250)	<0.001

Note: Low serum AG ratio was defined as quintile 1 and quintile 2 of serum AG ratio (<1.34).

Abbreviations: AG ratio, albumin-to-globulin ratio; BMI, body mass index; CI, confidence interval; CRP, C-reactive protein; eGFR, estimated glomerular filtration rate; OR, odds ratio; SBP, systolic blood pressure; WBC, white blood cell.
