# Supplementary material for: Predictive value of serum albumin-to-globulin ratio for incident chronic kidney disease: A 12-year community-based prospective study
Source: PLoS One. 2020 Sep 2;15(9):e0238421. doi: 10.1371/journal.pone.0238421 (PMC7467286; doi:10.1371/journal.pone.0238421)
Supplement: S2 Table — (PDF) [file pone.0238421.s002.pdf]

S2 Table. Crude and fully adjusted hazard ratios of serum AG ratio quintiles and other variables for CKD development

	Crude		[a]Fully adjusted	
	HR (95% CI)	P	HR (95% CI)	P
Serum AG ratio quintiles				
Q1 (<1.26)	1.608 (1.384-1.870)	<0.001	1.651 (1.406-1.938)	<0.001
Q2 (1.26 to <1.34)	1.328 (1.138-1.551)	<0.001	1.453 (1.235-1.708)	<0.001
Q3 (1.34 to <1.42)	1.253 (1.066-1.472)	0.01	1.419 (1.203-1.675)	<0.001
Q4 (1.42 to <1.55)	1.141 (0.972-1.340)	0.11	1.174 (0.998-1.381)	0.05
Q5 (≥1.55)	1 (reference)		1 (reference)	
Age (per 1 year increase)	1.092 (1.086-1.098)	<0.001	1.070 (1.062-1.077)	<0.001
Women (vs. men)	1.229 (1.118-1.352)	<0.001	1.475 (1.222-1.780)	<0.001
Education ≥7th grade (vs. ≤6th grade)	0.500 (0.455-0.550)	<0.001	1.098 (0.974-1.237)	0.13
Income ≥$1,000/m (vs. <$1,000/m)	0.512 (0.466-0.563)	<0.001	0.994 (0.888-1.113)	0.9
Smokers (vs. non-smokers)	1.131 (1.026-1.247)	0.01	1.222 (1.046-1.427)	0.01
DM (vs. non-DM)	2.261 (1.899-2.692)	<0.001	1.652 (1.353-2.017)	<0.001
Hypertension (vs. non-hypertensive)	2,193 (1.964-2.448)	<0.001	1.259 (1.113-1.425)	<0.001
CVD (vs. non-CVD)	1.982 (1.577-2.491)	<0.001	1.137 (0.902-1.435)	0.28
BMI (per 1 kg/m^2 increase)	1.046 (1.030-1.061)	<0.001	1.023 (1.007-1.040)	0.01
MAP (per 1 mmHg increase)	1.025 (1.021-1.028)	<0.001	1.007 (1.003-1.011)	0.001
Hemoglobin (per 1 g/dL increase)	0.993 (0.964-1.023)	0.66	1.022 (0.975-1.070)	0.37
Glucose (per 1 mg/dL increase)	1.006 (1.005-1.008)	<0.001	1.003 (1.001-1.006)	0.003
Total cholesterol (per 1 mg/dL increase)	1.004 (1.003-1.006)	<0.001	1.000 (0.999-1.002)	0.77
eGFR (per 1 ml/min/1.73 m^2 increase)	0.951 (0.948-0.955)	<0.001	0.959 (0.955-0.963)	<0.001

[a]Fully adjusted: adjusted for age, sex, education and income levels, smoking status, DM, hypertension, CVD, BMI, MAP, hemoglobin, serum glucose, total cholesterol, and baseline eGFR.

Abbreviations: AG ratio, albumin-to-globulin ratio; BMI, body mass index; CI, confidence interval; CKD, chronic kidney disease; CVD, cardiovascular disease; DM, diabetes mellitus; eGFR, estimated glomerular filtration rate; HR, hazard ratio; MAP, mean arterial pressure.
