# Supplementary material for: Predictive value of serum albumin-to-globulin ratio for incident chronic kidney disease: A 12-year community-based prospective study
Source: PLoS One. 2020 Sep 2;15(9):e0238421. doi: 10.1371/journal.pone.0238421 (PMC7467286; doi:10.1371/journal.pone.0238421)
Supplement: S3 Table — (PDF) [file pone.0238421.s003.pdf]

S3 Table. Crude and fully adjusted hazard ratios of serum AG ratio (per 0.2 decrement) and other variables for CKD development

	Crude		[a]Fully adjusted	
	HR (95% CI)	P	HR (95% CI)	P
Serum AG ratio (per 0.2 decrease)	1.166 (1.108-1.226)	<0.001	1.170 (1.109-1.234)	<0.001
Age (per 1 year increase)	1.092 (1.086-1.098)	<0.001	1.070 (1.062-1.078)	<0.001
Women (vs. men)	1.229 (1.118-1.352)	<0.001	1.482 (1.228-1.789)	<0.001
Education ≥7th grade (vs. ≤6th grade)	0.500 (0.455-0.550)	<0.001	1.098 (0.975-1.238)	0.12
Income ≥$1,000/m (vs. <$1,000/m)	0.512 (0.466-0.563)	<0.001	0.994 (0.888-1.112)	0.9
Smokers (vs. non-smokers)	1.131 (1.026-1.247)	0.01	1.212 (1.038-1.415)	0.02
DM (vs. non-DM)	2.261 (1.899-2.692)	<0.001	1.649 (1.350-2.014)	<0.001
Hypertension (vs. non-hypertensive)	2,193 (1.964-2.448)	<0.001	1.259 (1.113-1.424)	<0.001
CVD (vs. non-CVD)	1.982 (1.577-2.491)	<0.001	1.132 (0.897-1.428)	0.30
BMI (per 1 kg/m^2 increase)	1.046 (1.030-1.061)	<0.001	1.024 (1.007-1.040)	0.004
MAP (per 1 mmHg increase)	1.025 (1.021-1.028)	<0.001	1.007 (1.003-1.011)	0.001
Hemoglobin (per 1 g/dL increase)	0.993 (0.964-1.023)	0.66	1.022 (0.976-1.071)	0.35
Glucose (per 1 mg/dL increase)	1.006 (1.005-1.008)	<0.001	1.003 (1.001-1.006)	0.004
Total cholesterol (per 1 mg/dL increase)	1.004 (1.003-1.006)	<0.001	1.000 (0.999-1.002)	0.84
eGFR (per 1 ml/min/1.73 m^2 increase)	0.951 (0.948-0.955)	<0.001	0.959 (0.956-0.963)	<0.001

[a]Fully adjusted: adjusted for age, sex, education and income levels, smoking status, DM, hypertension, CVD, BMI, MAP, hemoglobin, serum glucose, total cholesterol, and baseline eGFR.

Abbreviations: AG ratio, albumin-to-globulin ratio; BMI, body mass index; CI, confidence interval; CKD, chronic kidney disease; CVD, cardiovascular disease; DM, diabetes mellitus; eGFR, estimated glomerular filtration rate; HR, hazard ratio; MAP, mean arterial pressure.
