# Supplementary material for: Predictive value of serum albumin-to-globulin ratio for incident chronic kidney disease: A 12-year community-based prospective study
Source: PLoS One. 2020 Sep 2;15(9):e0238421. doi: 10.1371/journal.pone.0238421 (PMC7467286; doi:10.1371/journal.pone.0238421)
Supplement: S4 Table — (PDF) [file pone.0238421.s004.pdf]

S4 Table. The number of participants with various kidney outcomes according to serum AG ratio quintiles

	n (%)			
	[a]Proteinuria	eGFR of <60 ml/min/1.73 m^2	eGFR decline of >50% from baseline	[b]Rapid decline of kidney function
Serum AG ratio quintiles				
Q1 (<1.26)	64 (3.9%)	397 (24.2%)	41 (2.5%)	629 (38.3%)
Q2 (1.26 to <1.34)	49 (3.0%)	348 (21.2%)	26 (1.6%)	519 (31.6%)
Q3 (1.34 to <1.42)	43 (2.9%)	286 (19.3%)	31 (2.1%)	454 (30.6%)
Q4 (1.42 to <1.55)	50 (3.1%)	281 (17.2%)	24 (1.5%)	421 (25.7%)
Q5 (≥1.55)	47 (2.8%)	241 (14.6%)	12 (0.7%)	334 (20.2%)

[a]Proteinuria was defined as urinary protein of more than 1+ on dipstick test. [b]Rapid decline of kidney function was defined as eGFR decline >3 ml/min/1.73 m^2 per year.

Abbreviations: AG ratio, albumin-to-globulin ratio; eGFR, estimated glomerular filtration rate.
