# Supplementary material for: Predictive value of serum albumin-to-globulin ratio for incident chronic kidney disease: A 12-year community-based prospective study
Source: PLoS One. 2020 Sep 2;15(9):e0238421. doi: 10.1371/journal.pone.0238421 (PMC7467286; doi:10.1371/journal.pone.0238421)
Supplement: S5 Table — (PDF) [file pone.0238421.s005.pdf]

S5 Table. Fully adjusted hazard ratios of serum AG ratio for CKD development after excluding underweight participants

	[a]Fully adjusted		
	n (%)	HR (95% CI)	P
Serum AG ratio quintiles			
Q1 (<1.26)	432 (26.7%)	1.667 (1.418-1.960)	<0.001
Q2 (1.26 to <1.34)	372 (23.1%)	1.463 (1.242-1.723)	<0.001
Q3 (1.34 to <1.42)	311 (21.4%)	1.424 (1.204-1.683)	<0.001
Q4 (1.42 to <1.55)	320 (19.9%)	1.188 (1.008-1.399)	0.04
Q5 (≥1.55)	270 (16.7%)	1 (reference)	
Serum AG ratio (per 0.2 decrease)		1.172 (1.110-1.237)	<0.001

Note: After excluding 144 (1.8%) underweight participants (BMI <18.5 kg/m^2), 7,913 participants were analyzed.

[a]Fully adjusted: adjusted for age, sex, education and income levels, smoking status, DM, hypertension, CVD, BMI, MAP, hemoglobin, serum glucose, total cholesterol, and baseline eGFR.

Abbreviations: AG ratio, albumin-to-globulin ratio; BMI, body mass index; CI, confidence interval; CKD, chronic kidney disease; CVD, cardiovascular disease; DM, diabetes mellitus; eGFR, estimated glomerular filtration rate; HR, hazard ratio; MAP, mean arterial pressure.
