# Supplementary material for: Predictive value of serum albumin-to-globulin ratio for incident chronic kidney disease: A 12-year community-based prospective study
Source: PLoS One. 2020 Sep 2;15(9):e0238421. doi: 10.1371/journal.pone.0238421 (PMC7467286; doi:10.1371/journal.pone.0238421)
Supplement: S6 Table — (PDF) [file pone.0238421.s006.pdf]

S6 Table. Fully adjusted hazard ratios of serum AG ratio for CKD development after excluding participants with high CRP concentrations

	[a]Fully adjusted		
	n (%)	HR (95% CI)	P
Serum AG ratio quintiles			
Q1 (<1.26)	397 (26.2%)	1.653 (1.402-1.949)	<0.001
Q2 (1.26 to <1.34)	353 (22.7%)	1.449 (1.227-1.711)	<0.001
Q3 (1.34 to <1.42)	302 (21.2%)	1.421 (1.200-1.683)	<0.001
Q4 (1.42 to <1.55)	308 (19.6%)	1.172 (0.993-1.384)	0.06
Q5 (≥1.55)	267 (16.8%)	1 (reference)	
Serum AG ratio (per 0.2 decrease)		1.171 (1.108-1.238)	<0.001

Note: We excluded 403 (5.0%) participants that their CRP concentrations were above 95 percentile (CRP >0.66 mg/L). After excluding 403 participants, 7,654 participants were analyzed.

[a]Fully adjusted: adjusted for age, sex, education and income levels, smoking status, DM, hypertension, CVD, BMI, MAP, hemoglobin, serum glucose, total cholesterol, and baseline eGFR.

Abbreviations: AG ratio, albumin-to-globulin ratio; BMI, body mass index; CI, confidence interval; CKD, chronic kidney disease; CVD, cardiovascular disease; DM, diabetes mellitus; eGFR, estimated glomerular filtration rate; HR, hazard ratio; MAP, mean arterial pressure.
