# Supplementary material for: Predictive value of serum albumin-to-globulin ratio for incident chronic kidney disease: A 12-year community-based prospective study
Source: PLoS One. 2020 Sep 2;15(9):e0238421. doi: 10.1371/journal.pone.0238421 (PMC7467286; doi:10.1371/journal.pone.0238421)
Supplement: S7 Table — (PDF) [file pone.0238421.s007.pdf]

S7 Table. Fully adjusted hazard ratios of serum AG ratio for CKD development after excluding participants with eGFR of <70 ml/min/1.73 m^2

	[a]Fully adjusted		
	n (%)	HR (95% CI)	P
Serum AG ratio quintiles			
Q1 (<1.26)	387 (24.9%)	1.620 (1.359-1.930)	<0.001
Q2 (1.26 to <1.34)	333 (21.2%)	1.370 (1.147-1.636)	<0.001
Q3 (1.34 to <1.42)	268 (19.1%)	1.321 (1.100-1.585)	<0.001
Q4 (1.42 to <1.55)	262 (17.2%)	1.143 (0.954-1.369)	0.15
Q5 (≥1.55)	223 (14.7%)	1 (reference)	
Serum AG ratio (per 0.2 decrease)		1.162 (1.097-1.232)	<0.001

Note: After excluding 487 participants with eGFR of <70 ml/min/1.73 m^2, 7,570 participants were analyzed.

[a]Fully adjusted: adjusted for age, sex, education and income levels, smoking status, DM, hypertension, CVD, BMI, MAP, hemoglobin, serum glucose, total cholesterol, and baseline eGFR.

Abbreviations: AG ratio, albumin-to-globulin ratio; BMI, body mass index; CI, confidence interval; CKD, chronic kidney disease; CVD, cardiovascular disease; DM, diabetes mellitus; eGFR, estimated glomerular filtration rate; HR, hazard ratio; MAP, mean arterial pressure.
