# Supplementary material for: Predictive value of serum albumin-to-globulin ratio for incident chronic kidney disease: A 12-year community-based prospective study
Source: PLoS One. 2020 Sep 2;15(9):e0238421. doi: 10.1371/journal.pone.0238421 (PMC7467286; doi:10.1371/journal.pone.0238421)
Supplement: S8 Table — (PDF) [file pone.0238421.s008.pdf]

S8 Table. Reclassification table and categorical NRI of base model, serum AG ratio, WBC, and CRP for CKD development

	Base model + AG ratio				Base model + WBC				Base model + CRP			
[a]Base model	<10%	10-50%	>50%	% Reclassified	<10%	10-50%	>50%	% Reclassified	<10%	10-50%	>50%	% Reclassified
Participants without outcome												
<10%	2,442	147	0	6	2,541	48	0	2	2,583	6	0	0
10-50%	207	3,180	39	7	45	3,373	8	2	4	3421	1	0
>50%	0	45	265	15	0	15	295	5	0	0	310	0
Participants with outcome												
<10%	141	18	0	11	156	3	0	2	158	1	0	1
10-50%	15	1,104	55	6	4	1,158	12	1	1	1,171	2	0
>50%	0	29	370	7	0	7	392	2	0	2	397	1
Categorical NRI (SEM; 95% CI)	0.027 (0.013-0.041)				0.003 (-0.004-0.010)				-0.0004 (-0.003-0.003)			

[a]Base model included age, sex, education and income levels, smoking status, diabetes, hypertension, cardiovascular disease, body mass index, mean arterial pressure, hemoglobin, serum glucose, total cholesterol, and baseline eGFR.

Abbreviations: AG ratio, albumin-to-globulin ratio; CI, confidence interval; CKD, chronic kidney disease; CRP, C-reactive protein; eGFR, estimated glomerular filtration rate; NRI, net reclassification index; SEM, standard error of the means; WBC, white blood cell.
