# Supplementary figures and images for: Predictive value of serum albumin-to-globulin ratio for incident chronic kidney disease: A 12-year community-based prospective study
Source: PLoS One. 2020 Sep 2;15(9):e0238421. doi: 10.1371/journal.pone.0238421 (PMC7467286; doi:10.1371/journal.pone.0238421)

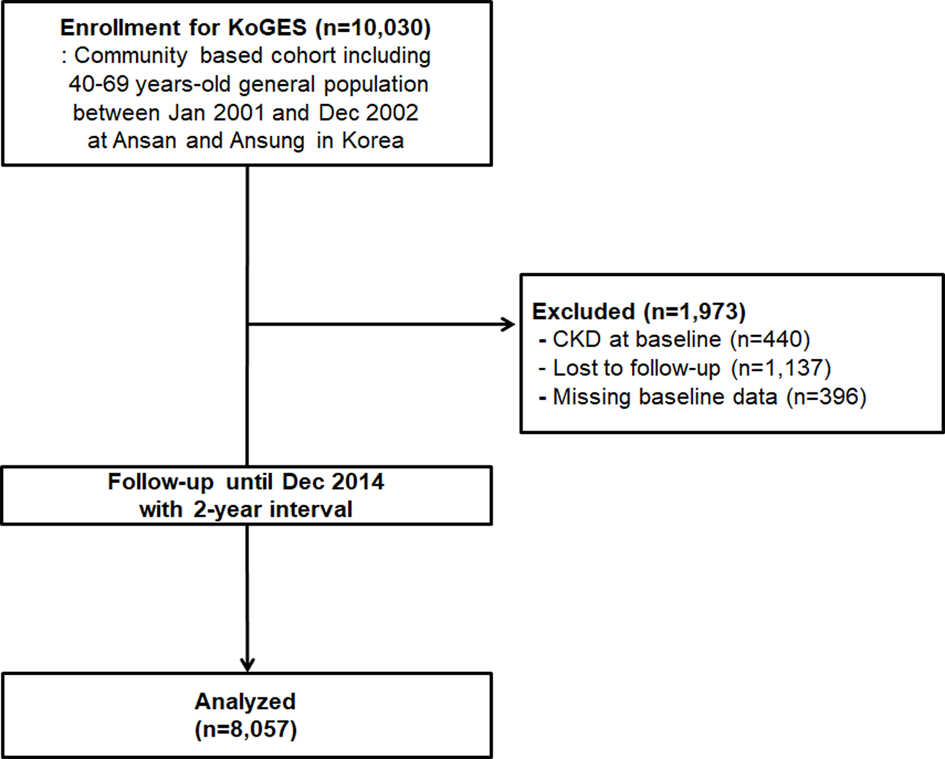

Supplement: S1 Fig — (TIF) [file pone.0238421.s009.tif]
